# Domesticated *Argania spinosa* in Eastern Morocco: HPLC-DAD/GC-MS Chemical Profiling, Antioxidant and Antidiabetic Activities, and Network Pharmacology-Guided Molecular Docking

**DOI:** 10.3390/ijms27177964

**Published:** 2026-09-07

**Authors:** Salah-eddine Azizi, Nour Elhouda Daoudi, Mohammed Roubi, Ilyass Alami Merrouni, Adha Fauzi Hendrawan, Mohamed Bnouham, Abdelbasset Berrichi, Mohammed Dalli, Bouchra Legssyer, Nadia Gseyra

**Affiliations:** 1 Higher Institute of Nursing Professions and Health Techniques (ISPITSO), Oujda 60000, Morocco; n.daoudi@ump.ac.ma (N.E.D.); alami.ilyass.90@gmail.com (I.A.M.); 2 Laboratory of Pharmacology, Genomics, Environment and Health (PHARGENES Lab), Faculty of Sciences, Mohammed First University, Boulevard Mohamed VI, B.P.717, Oujda 60000, Morocco; mohammed.roubi1@ump.ac.ma (M.R.); mbnouham@yahoo.fr (M.B.); ngseyra@hotmail.com (N.G.); 3 Faculty of Medicine, Public Health, and Nursing, Universitas Gadjah Mada, Yogyakarta 55281, Indonesia; adhafauzihendrawan2004@mail.ugm.ac.id; 4 Laboratory for Agricultural Production Improvement, Biotechnology and Environment, Faculty of Sciences, Mohammed First University, Boulevard Mohamed VI, B.P.717, Oujda 60000, Morocco; abdel20759@yahoo.fr (A.B.); legssyerb@hotmail.com (B.L.); 5 Life and Drug Sciences Research Laboratory, Faculty of Medicine and Pharmacy of Oujda, Mohammed First University, Oujda 60000, Morocco

**Keywords:** argan tree, eastern Morocco, antidiabetic, antioxidant, *Argania spinosa*

## Abstract

The argan tree (*Argania spinosa*) is an endemic Moroccan species known for its primary product, argan oil, which possesses exceptional nutritional and medicinal properties. The current study aimed to evaluate and compare the antidiabetic and antioxidant activities of argan oil obtained from the introduced and native argan tree in eastern Morocco, to analyze its chemical composition using HPLC-DAD and GC-MS, and to investigate the molecular mechanisms behind the obtained pharmacological activities through an in silico pharmacological networking and molecular docking study. The results revealed that argan oil from all three regions of Morocco (Oujda, Agadir, and Chouihya) is rich in oleic and linoleic acids as major constituents, along with the presence of significant tocopherols. Regarding the antioxidant assays, including DPPH radical scavenging and iron-reducing power tests, argan oil from Oujda exhibited the highest activity, with the lowest IC_50_ values of 15.25 ± 0.022 mg/mL and 28.5 ± 1.7 mg/mL, respectively. Concerning the antidiabetic activity, we found that oil from Chaouihya showed the strongest α-amylase inhibition, while Oujda oil had the highest antiglycation activity, indicating that even introduced argan trees retain potent bioactivity. The results of the in silico investigation suggested that tocopherols may contribute to the antioxidant and antidiabetic potential of argan oil, showing predicted antioxidant activity (Pa = 0.843–0.967) and favorable binding affinities toward iNOS (ΔG = −9.3 kcal mol^−1^) and α-glucosidase (ΔG = −8.2 kcal mol^−1^). The identified fatty acids also showed predicted insulin-promoting activity (Pa = 0.59–0.75) and moderate enzyme-binding potential. Pharmacological network analysis identified 51 shared genes associated with antioxidant, antidiabetic, and argan-related targets, with enrichment of the AGE–RAGE signaling pathway. These computational findings provide possible molecular associations that may help explain the observed biological activities, although they remain predictive and require experimental validation. Overall, the in silico analysis suggests that tocopherols could be among the contributors to the multi-target profile of *Argania spinosa* oil, while fatty acids may provide complementary effects related to glycemic regulation.

## 1. Introduction

Diabetes mellitus is a long-lasting condition resulting from insufficient insulin production by the pancreas or ineffective use of the produced insulin in the body. Insulin is crucial for monitoring blood sugar levels. When diabetes is not properly managed, it often leads to hyperglycemia, an elevated concentration of sugar in the blood, causing severe damage to various organ systems over time, particularly affecting nerves and blood vessels [1]. Insulin-dependent or juvenile diabetes, which is also called type 1 diabetes, is characterized by a lack of insulin production and necessitates daily insulin injections. In contrast, type 2 diabetes, previously known as non-insulin-dependent or mature-onset diabetes, arises from the body’s improper utilization of insulin [2].

Chronic hyperglycemia is associated with the development of several complications, including macroangiopathy and microangiopathy, which affect large and small blood vessels, respectively [3]. One of the key mechanisms underlying these complications is the glycation of proteins, such as hemoglobin, which forms advanced glycation end products (AGEs) that contribute to tissue damage and dysfunction [4].

From a physiological perspective, the gastrointestinal tract contains a diverse range of digestive enzymes, including α-amylase. This enzyme plays a significant role in breaking down starch by cleaving the α-1,4 glycosidic bond, leading to the production of maltose and glucose. There are two primary types of α-amylase enzymes: salivary amylase, found in saliva, and pancreatic amylase, which continues starch digestion in the small intestine. Inhibiting α-amylase is seen as a crucial target for controlling postprandial blood glucose levels, particularly in diabetes management [5]. Based on the current understanding of the pathophysiology of insulin resistance and type 2 diabetes, multiple pharmacological and non-pharmacological interventions have been developed with the aim of improving glycemic control and preventing diabetic complications. Among others, the use of functional foods and their bioactive components has been considered as a new approach to the prevention and management of diabetes and its complications [4].

*Argania spinosa* is a native plant of Morocco from the Sapotaceae family and covers a significant area of 80,000 Ha [6,7,8]. This plant is highly condensed in southern Morocco and is considered a UNESCO-protected biosphere. Also, it exists as a relic in the northern regions such as Beni-Snassen, Oued-Cherrat, and Plaine Bou-Areg, due to favorable bioclimatic conditions that support its growth [9]. Argan oil, extracted from the plant’s almonds, is renowned worldwide for its nutritional value and numerous health benefits [10,11]. This oil is rich in tocopherols, unsaturated fatty acids, and phenolic compounds, contributing to its medicinal virtues [12]. Notably, the leaves, pulp, and flowers of the plant are also active in traditional medicine, containing bioactive components like rutin, myricitrin, and hyperoside [13]. Traditional practices in southern Morocco have long utilized these parts to treat various illnesses. Argan almonds are used in powder form via external application for the treatment of eczema, burns and hair revitalization, and through oral administration for the treatment of diabetes and general nutrition [14,15]. The fixed oil is still currently used alone or often flavored with essential oils (rosemary, black cumin, etc.). Also, it is used per os administration for the treatment of rheumatism, hypercholesterolemia, lung infections, gastrointestinal problems and the prevention of cardiovascular disease, and as an antioxidant and hepatoprotective agent [16]. Scientific research on *Argania spinosa* has corroborated these traditional beliefs, confirming its diverse medicinal properties [16,17]. Conducted studies in the field have shown its effectiveness in areas such as antidiabetic [18] and anti-inflammatory effects, antihypertensive properties, and antioxidant activity [18,19]. This large spectrum of biological benefits positions Argan as a promising candidate in the field of alternative medicine [20].

The aim of the current study is to evaluate and compare the antioxidant and antidiabetic properties of *Argania spinosa* fixed oil of both introduced and native argan trees from Oujda city and the Chouhiya region, respectively. This study stands out for its original approach, aiming to expand knowledge on argan oil from less-studied regions, in contrast to the widely studied argan from Agadir, which serves as a reference model. To better understand the pharmacological effects of our samples, the chemical composition of the oil was elucidated using HPLC-DAD and GC-MS.

## 2. Results

### 2.1. Chemical Composition of Argania spinosa Fixed Oil

Evaluation of the chemical composition of the methyl ester produced from argan oil by GC-MS showed the presence of four main fatty acids: oleic acid, linoleic acid, palmitic acid and stearic acid (ranked in order of abundance). Argan oil from Chouihiya was the richest in oleic and linoleic acids at 51.58% and 31.38%, respectively, followed by argan oil from Oujda at 49.31% and 30.54% (Table 1, Figures S1 and S2). Furthermore, tocopherol composition analysis showed the presence of three elements, γ-tocopherols, α-tocopherol, and δ-tocopherols, ranked in order of abundance. Total tocopherol content ranged from 531.94 mg/kg in argan oil from Chouihiya to 452.99 mg/kg in argan oil from Agadir. Finally, the evaluation of the physicochemical quality of the oils showed that the values are below the limits set by the Moroccan standard relating to the quality of argan oil.

**Figure 1:**
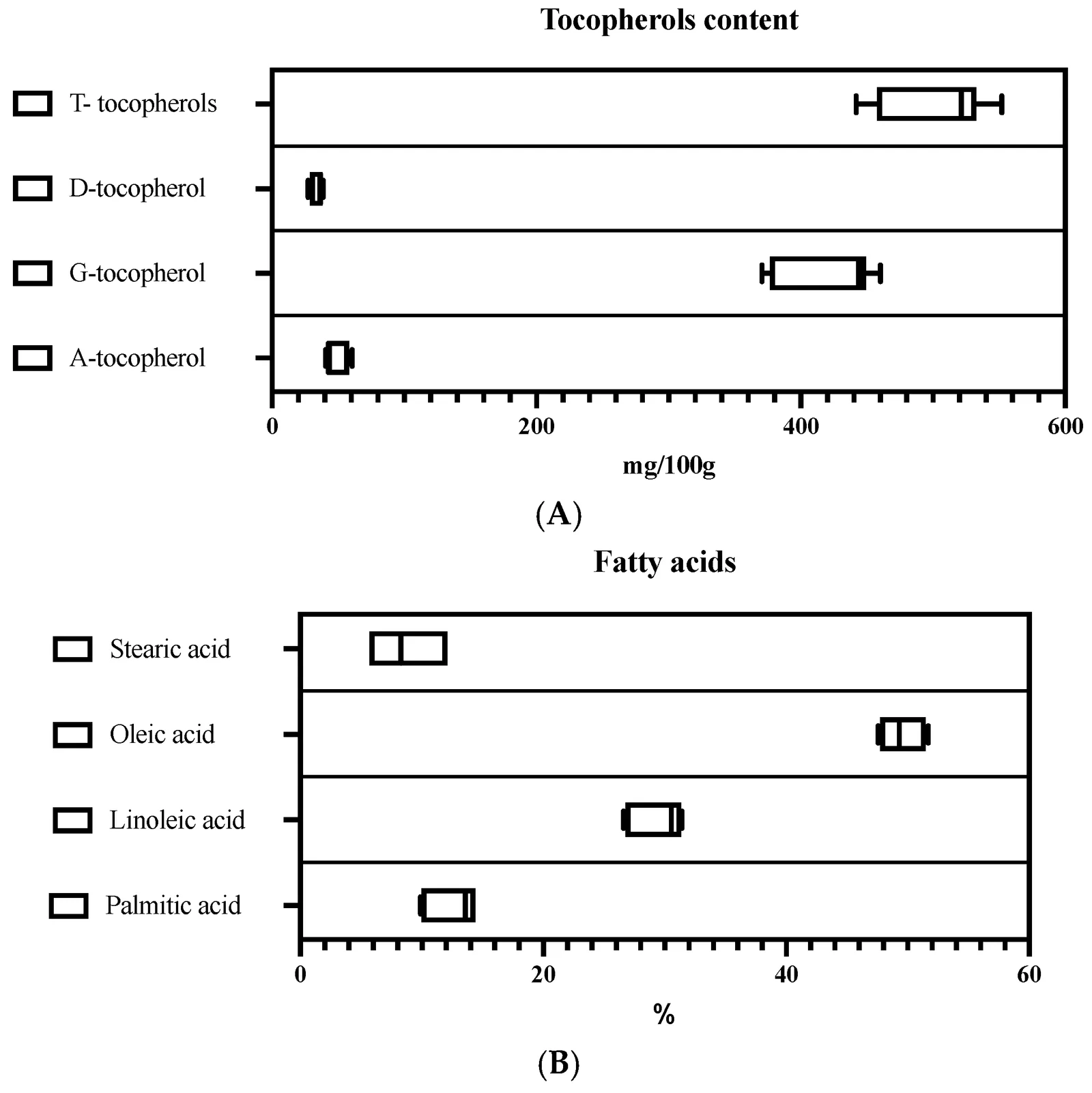

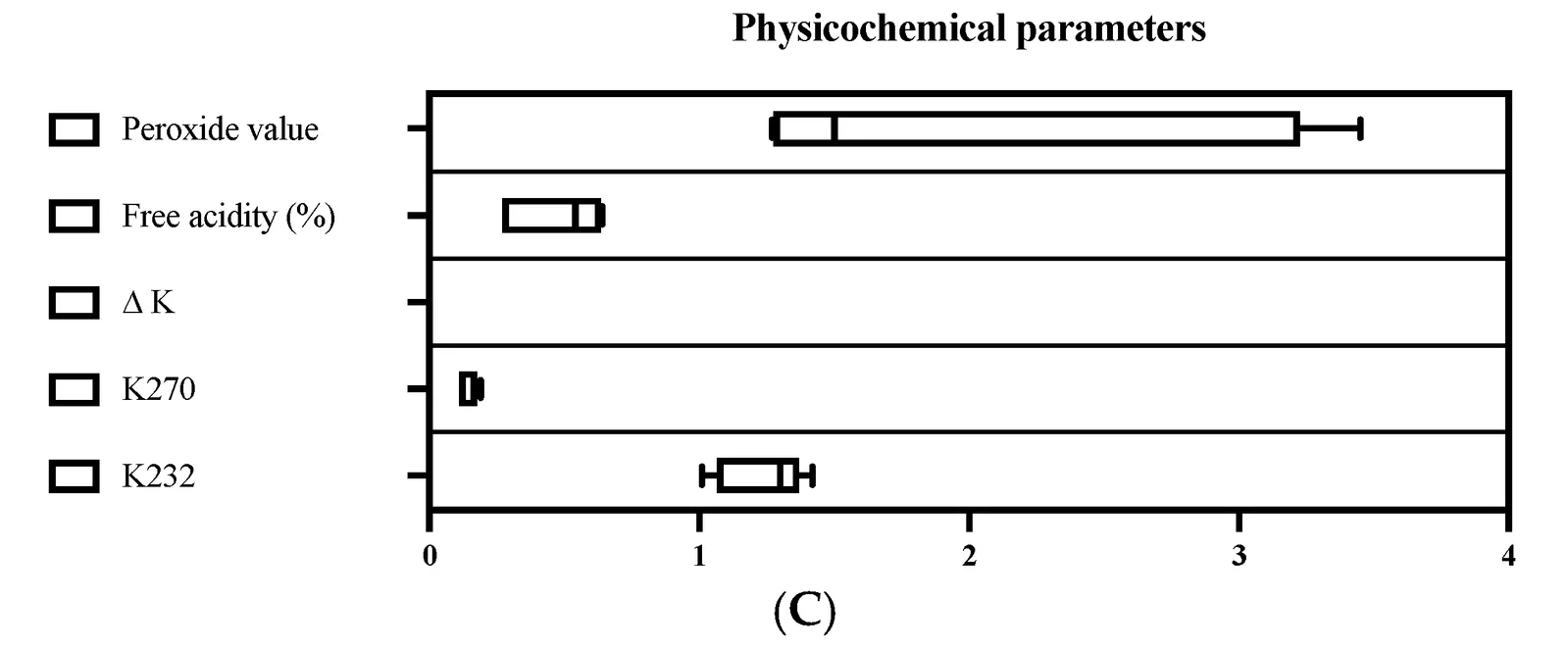
Boxplots showing the tocopherol content (**A**), fatty acid composition (**B**), and physicochemical parameters (**C**) of argan oils from three different origins. For each origin, measurements were performed in triplicate (n = 3 per origin). In each boxplot, the central line represents the median, while the lower and upper boundaries of the box represent the 25th and 75th percentiles, respectively, corresponding to the interquartile range (IQR). The whiskers indicate the range of the observed values.

The distribution of total tocopherols exhibits significant variability, with a relatively wide boxplot (Figure 1). This indicates that total vitamin E content varies greatly from one sample to another.

The δ-tocopherol shows a more homogeneous distribution, with a relatively compact box and low-value dispersion. The γ-tocopherol displays more pronounced variability, with a wider box and extreme values.

Oleic acid has a relatively concentrated distribution, with a moderately wide box, indicating some variability among samples. Linoleic acid exhibits a more heterogeneous distribution, with a broader box and detected outliers. This suggests more pronounced variability between samples.

The distribution of the peroxide value shows significant variability among samples. The range of the whiskers indicates a wide dispersion of values, suggesting notable differences in lipid oxidation across samples. The presence of outliers could indicate that certain samples have undergone more advanced oxidation, possibly due to prolonged exposure to oxygen or inadequate storage conditions.

Free acidity has a tighter distribution compared to the peroxide value, suggesting relative homogeneity among the samples. However, the presence of extreme values indicates that some samples have significantly higher acidity levels.

These results highlight notable differences between samples for certain physicochemical parameters. The presence of outliers for the peroxide value and free acidity suggests that some samples have undergone greater degradation, which could be attributed to specific storage or production conditions. Absorbance indices confirm these observations, particularly for G 270, which is a good indicator of secondary lipid oxidation.

### 2.2. In Silico Analysis of Argan Oil

The results from HPLC–DAD and GC–MS resolved eight constituents of argan oil: four major fatty acids (palmitic, linoleic, oleic, stearic) and a tocopherol fraction (α, γ, δ; plus total tocopherols), with the molecular formulae/masses shown in Table 2. These compounds were advanced to activity prediction, network analysis, and docking to test whether chemical composition aligns with antioxidant and antidiabetic mechanisms. Structure–activity screening using PASS predicted that tocopherols had the highest antioxidant potential (Pa = 0.843–0.967), whereas fatty acids showed moderate antioxidant scores (Pa = 0.222–0.314) but uniquely carried enzyme-level activities (superoxide dismutase and catalase inhibition, Pa ≈ 0.81–0.91; α/β-amylase inhibition, Pa ≈ 0.30–0.59). Fatty acids also exhibited higher probabilities for insulin-promoting effects (Pa ≈ 0.59–0.75) than tocopherols (Pa ≈ 0.28–0.31). All compounds passed Lipinski filters but triggered Pfizer/GSK structural alerts; predicted acute toxicity was generally low for tocopherols (LD_50_ 5000 mg kg^−1^; class 5) and variable across fatty acids (class 6 to class 2) (Table 3). Guided by these computational predictions, we then explored whether known molecular targets may connect these constituents to disease-relevant pathways.

**Figure 2:**
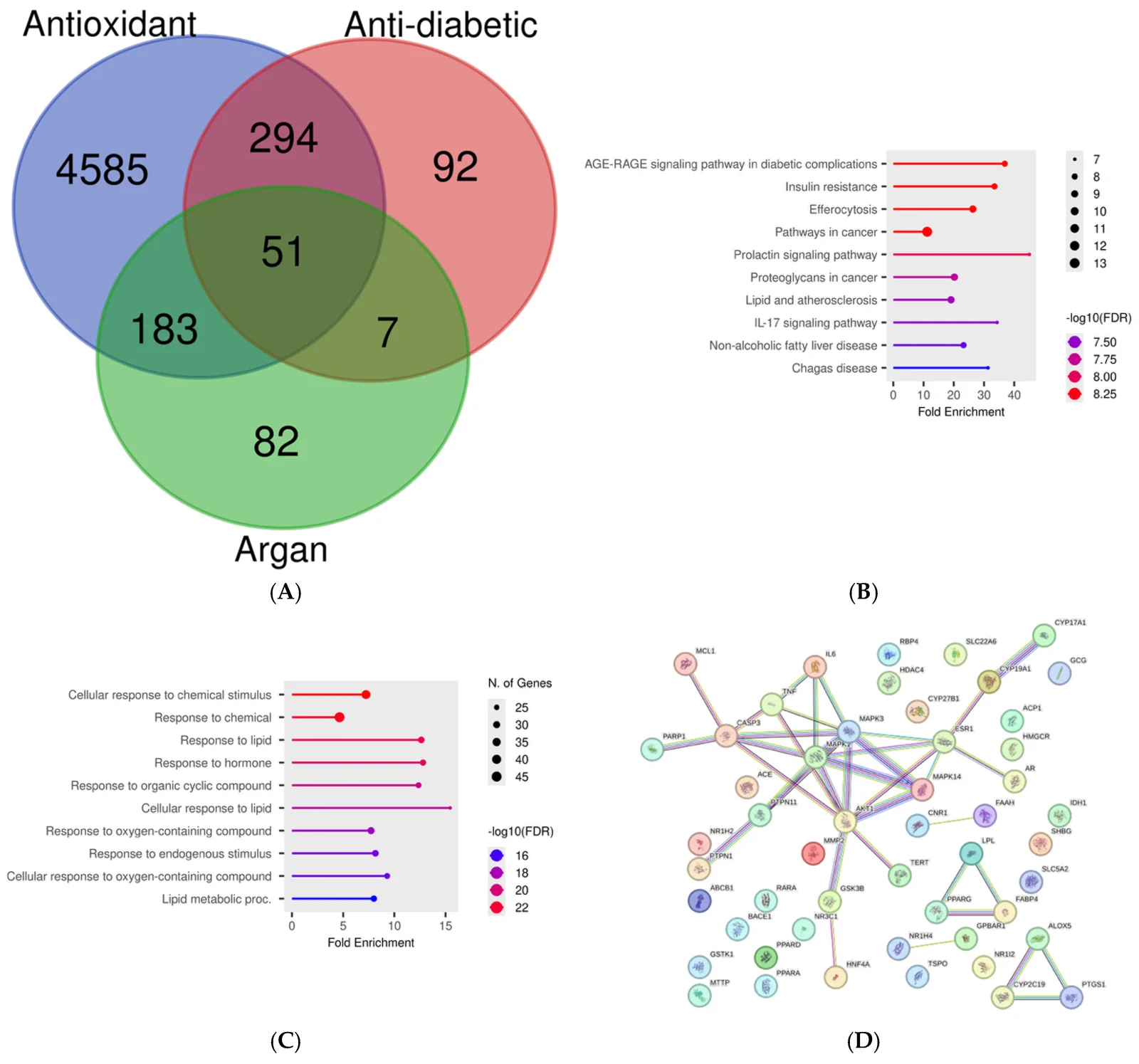
Network pharmacology of *Argania spinosa* in antioxidant and antidiabetic: (**A**) shared target overlap (Venn), (**B**) enriched KEGG pathways, (**C**) enriched GO Biological Process, and (**D**) PPI network linking common targets.

Integrating targets annotated for antioxidant activity, antidiabetic activity, and *Argania spinosa* revealed a non-random overlap of 51 shared genes (with additional pairwise overlaps of 294, 183, and 7; set-unique genes 4585, 92, and 82; Figure 2A). Enrichment analysis of the intersecting set highlighted metabolic–inflammatory axes—AGE–RAGE signaling, insulin resistance, efferocytosis, lipid/cancer pathways, IL-17 signaling, NAFLD, and others (q ≤ 10^−7.5^; Figure 2B). Consistently, GO Biological Processes emphasized responses to chemical/lipid/hormone stimuli and lipid metabolism (q ≤ 10^−16^; Figure 2C). A PPI map revealed dense interconnections among signaling and metabolic proteins (Figure 2D), providing potential molecular associations related to the predicted activities shown in Table 3.

To further explore the potential interactions suggested by the network analysis, we docked all eight constituents against representative antioxidant and glycemic-control targets. Tocopherols showed the most favorable predicted binding affinities overall: δ-tocopherol led for iNOS (ΔG = −9.3 kcal mol^−1^) and α-glucosidase (−8.2), while α/γ-tocopherol ranked best for α-amylase (−8.6/−8.3), outperforming acarbose (−7.6 for amylase; −7.0 for glucosidase) and trolox against iNOS (−8.5). Fatty acids bound moderately (e.g., palmitic −6.6/−5.5/−6.2/−5.4; linoleic −7.0/−5.6/−6.1/−4.8), and at TCF7L2, the controls slightly exceeded tocopherols (acarbose −6.8, trolox −6.2 vs. tocopherols −6.0 to −5.7) (Table 4). These target-level data provide computational support for potential interactions between the identified constituents and the selected targets, with tocopherols, particularly δ-tocopherol, showing relatively favorable predicted binding affinities. Fatty acids also showed predicted interactions with several targets, suggesting that they may contribute to the overall predicted molecular profile of argan oil in Table 2. The computational analysis suggests that tocopherols may contribute to the multi-target pharmacological profile of *Argania spinosa* oil, while fatty acids may provide complementary effects; however, the contribution of other uncharacterized minor constituents cannot be excluded.

### 2.3. Antioxidant Activity of Argan Fixed Oil

#### Free Radical Scavenging Activity (DPPH)

**Figure 3:**
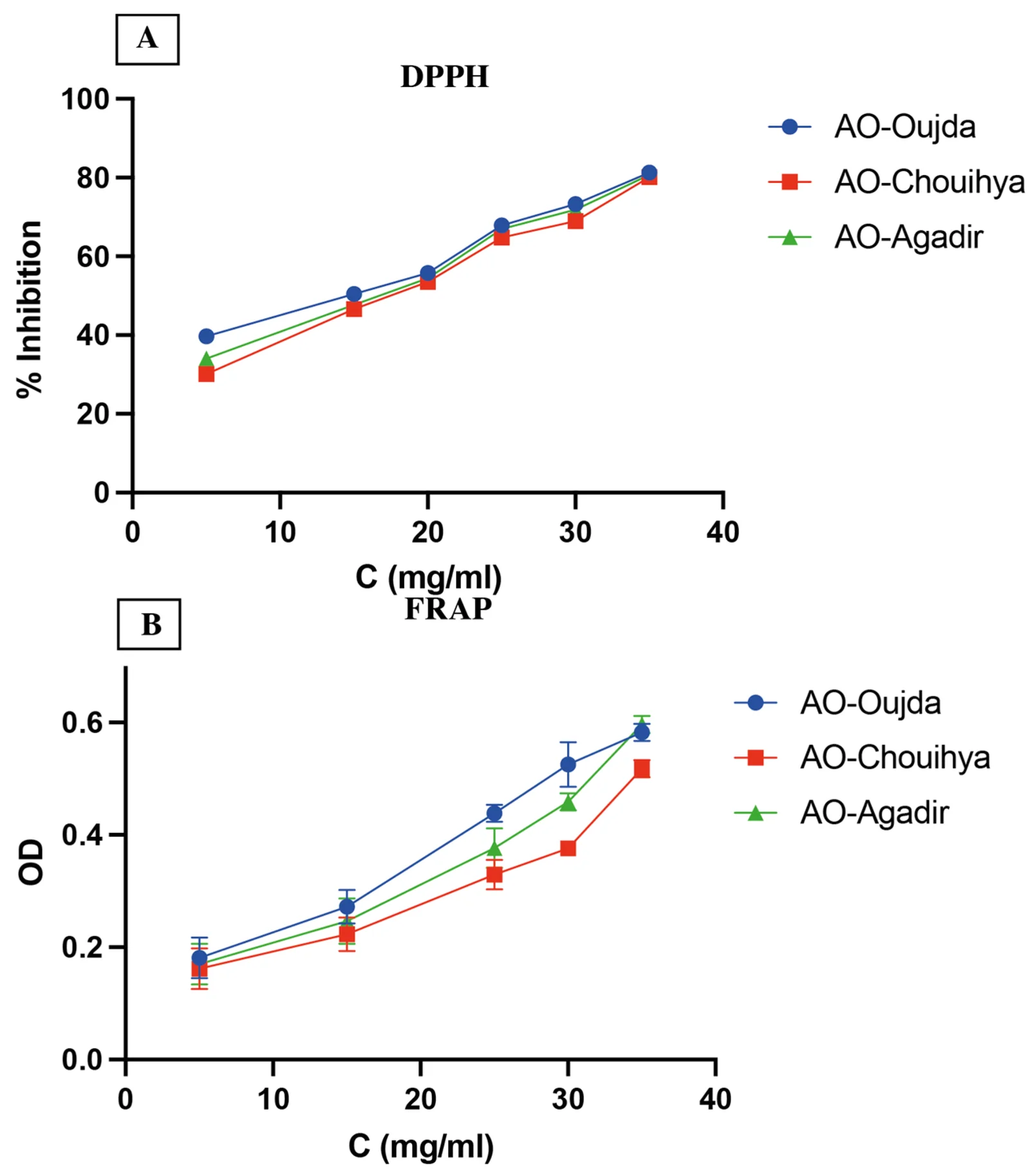
(**A**) Evolution of free radical scavenging activity of *Argania spinosa* fixed oils against stable radical DPPH•. (**B**) Evolution of optical density as a function of oil concentration.

Figure 3A shows that the percentage of inhibition increases in a concentration-dependent manner, reaching an inhibition rate of over 80% at a concentration of 35 mg/mL for all tested oils. Argan oil from Oujda gave the highest value of 81.27%, while argan oil from Chouihiya recorded the lowest value of 80.73%. At this concentration, ANOVA showed that there was no significant difference between the three studied fixed oils (*p* > 0.05).

The IC_50_ values are reported in the table below (Table 5), where the lowest value was recorded for argan oil from Oujda with a value of 15.25 ± 0.022 mg/mL, while the highest value was recorded for argan oil from Chouihya. Statistical analysis showed that there was a highly significant difference between the different oils tested (*p* < 0.0001).

Ferric reducing power assay:

Concerning the evaluation of the reducing power of ferric iron to ferrous iron, the results illustrated in Figure 3B show that optical density increases as a function of concentration, reaching a maximum of 0.596 at a concentration of 35 mg/mL. The highest value was recorded for argan oil from Agadir, followed by Oujda 0.582 and, finally, Chouihya 0.517. Statistical analysis showed that there was a highly significant difference between the different oils in terms of maximum concentration.

The IC_50_ value, which is considered to be the concentration that results in an optical density of 0.5 (Table 6), indicated that the lowest value was recorded for argan oil from Oujda 28.5 ± 1.7 mg/mL, followed by oil from Agadir, and finally Chouihya with values of 31.3 ± 0.75 mg/mL and 34.2 ± 0.28 mg/mL, respectively. Statistical analysis showed that there was a significant to highly significant difference between the different oils tested (*p* < 0.05–0.01).

### 2.4. Antidiabetic Activity of Argan Oil

#### 2.4.1. In Vitro Inhibitory Activity of α-Amylase

**Figure 4:**
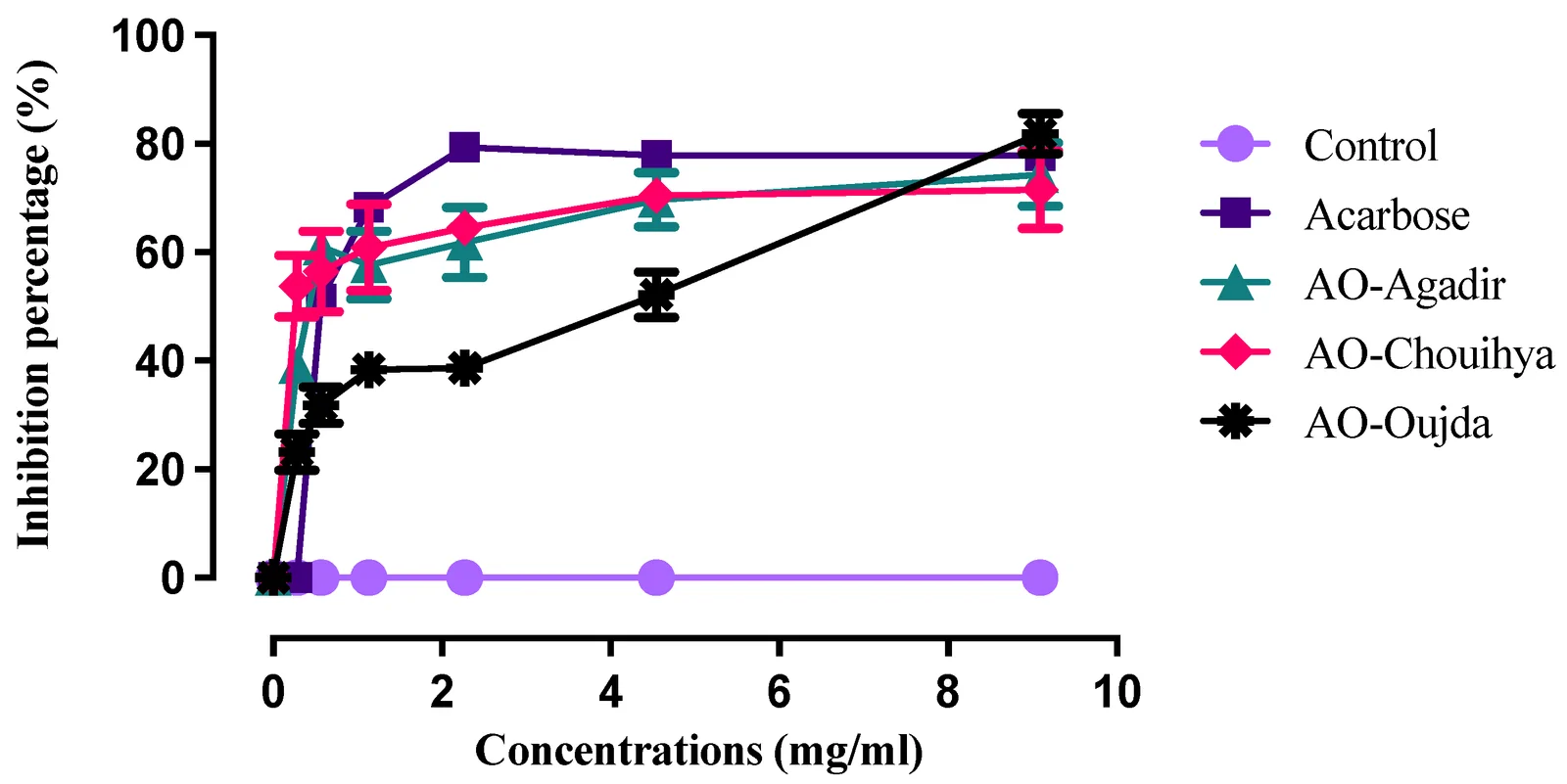
Evolution of α-amylase inhibitory activity as a function of oil concentration.

The evolution of α-amylase inhibitory activity was increased by the increasing oil concentration, as shown in Figure 4. The results showed that argan oil originating from Oujda gave the highest inhibitory activity, with a rate of 81.8% at a concentration of 9 mg/mL, while the same concentration of oil originating from Chouihya and Agadir gave inhibitory activities of 71.50 and 74.31%, respectively. On the other hand, acarbose used as a positive control gave a maximum inhibition of 77.80%.

Chouihya argan oil showed the strongest α-amylase inhibition, needing the lowest concentration to achieve 50% inhibition, followed by that from Agadir 0.709 mg/mL, while argan oil from Oujda gave the highest IC_50_ (3.66 mg/mL) (Table 7). Statistical analysis showed that there was no statistically significant difference between acarbose and argan oil from Chouihya and Agadir (*p* > 0.05), while there was a highly significant difference with argan oil from Oujda *p* < 0.01.

#### 2.4.2. In Vitro Hemoglobin Glycation Inhibition Assay

**Figure 5:**
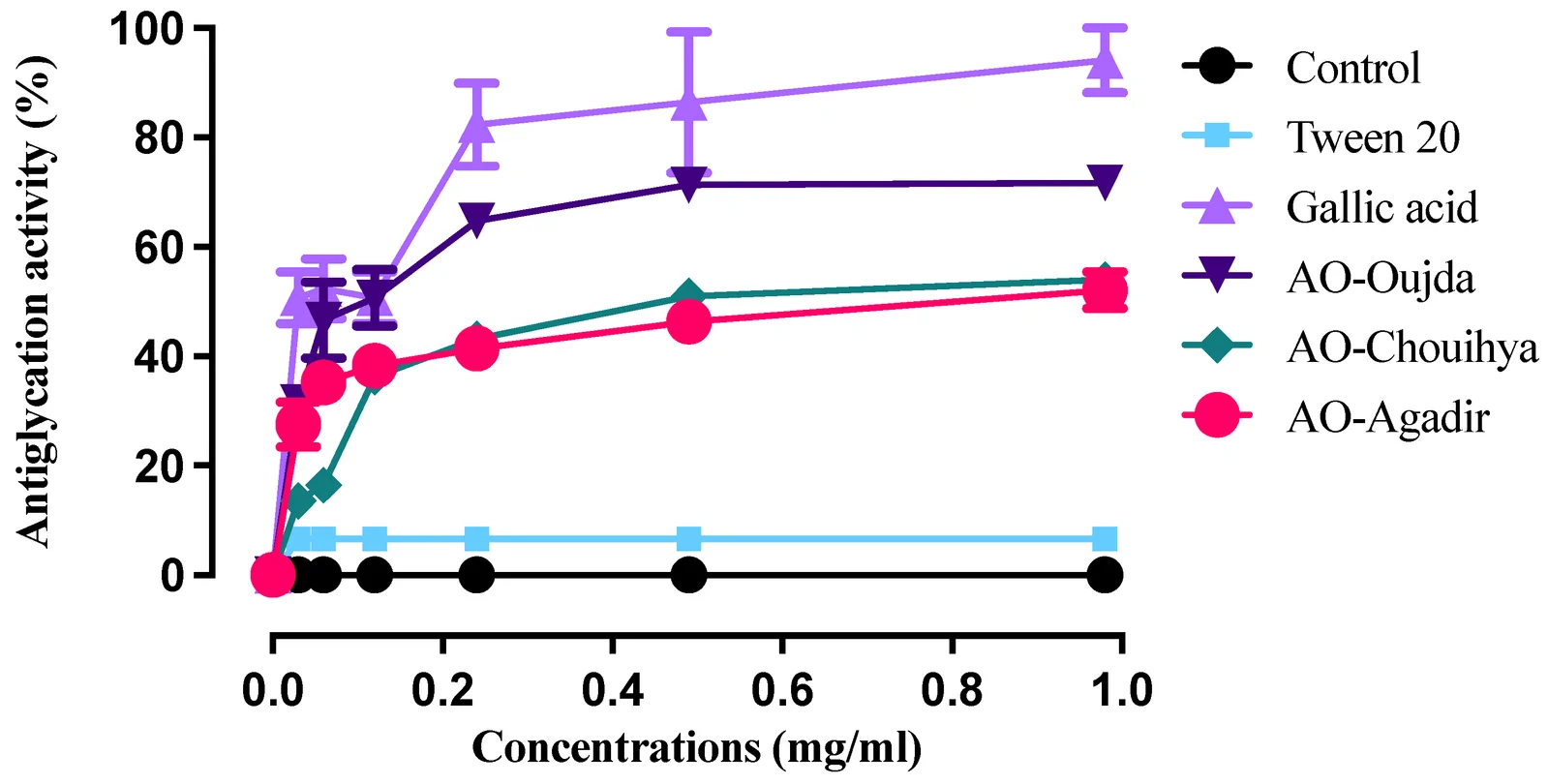
In vitro hemoglobin glycation inhibition by *Argania spinosa* fixed oils.

Figure 5 shows the evolution of hemoglobin glycation inhibition activity increased with the increase in oil concentration. The results showed that argan oil from Oujda revealed the highest value, with an inhibition rate of 70% at a concentration of 1 mg/mL, while the lowest value was recorded for argan oil from Agadir and Chouihya, with an inhibition rate of 50% at a concentration of 1 mg/mL. On the other hand, gallic acid used as a control showed much higher inhibitory activity than the different oils, with an inhibition rate reaching 95% at a concentration of 1 mg/mL.

Moreover, Table 8 shows the IC_50_ hemoglobin glycation by the different argan oils and by the standard. The results showed that argan oil from Oujda and gallic acid used as a positive control gave the lowest IC_50_ values of 0.097 mg/mL and 0.07 mg/mL, respectively, with no statistically significant difference between the two (*p* > 0.05). On the other hand, argan oil from Agadir gave the highest IC_50_ 0.77 mg/mL, with a highly significant difference from the standard used (*p* < 0.001).

**Figure 6:**
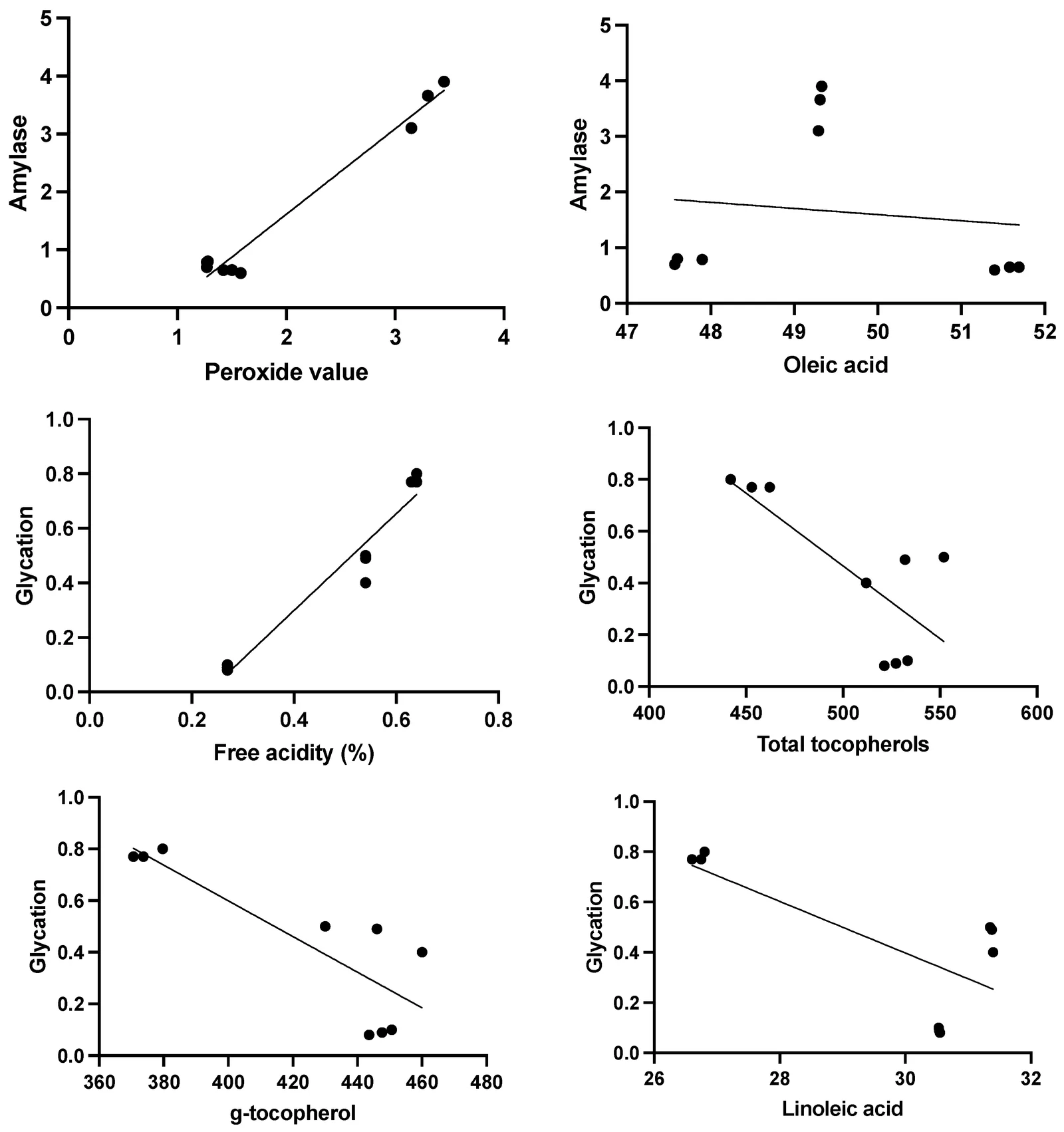
Statistical correlation between the chemical composition of oils and target proteins.

Statistical correlation:

The results of statistical correlations between the antioxidant activity, the antidiabetic activity, and the chemical composition of the oils are tabulated in Table 9 and Figure 6. Overall, the correlation coefficients were moderate to high in magnitude, but none reached statistical significance (*p* > 0.05 in all cases), and the results should, therefore, be interpreted as preliminary trends rather than established relationships.

A negative correlation was observed between palmitic acid content and the IC50 values of the DPPH radical scavenging assay (r = −0.948, *p* = >0.05) and the FRAP assay (r = −0.942, *p* > 0.05), suggesting a possible association between higher palmitic acid content and lower IC50 in both methods, whereas a positive correlation was found between α-tocopherol content and the IC50 obtained by both methods (r = 0.898–0.891, *p* > 0.05), suggesting that higher α-tocopherol levels may be associated with higher IC50 values, i.e., comparatively lower antioxidant activity.

Regarding the antidiabetic activities assessed by the two methods, correlations of varying strength were observed between the different parameters, none of which were statistically significant (*p* > 0.05). The IC50 of α-amylase inhibition showed a positive association with palmitic acid, stearic acid, and γ-tocopherol content. For hemoglobin glycation inhibition, IC50 was positively associated with α-tocopherol content and negatively associated with palmitic acid content, hinting at a possible link between higher palmitic acid content and greater inhibitory potential on hemoglobin glycation. Given the lack of statistical significance and the limited sample size, these associations warrant confirmation in future studies with larger sample sizes.

## 3. Discussion

Regarding the chemical composition of argan oil extracted from the three genotypes under examination, GC-MS analysis of methyl esters revealed the presence of four primary fatty acids across the tested samples. These compounds are primarily composed of oleic acid, with lower amounts of linoleic, palmitic, and stearic acids. These obtained results align with those reported in previous studies [21], which have confirmed that oleic acid is an abundant fatty acid in argan oil (43.1–57.0%), followed by linoleic acid (29.30–36.00%), palmitic acid (5.00–15.0%) and finally stearic acid (4.30–12%). In parallel, all-natural tocopherols are derived from plant sources, in which they are concentrated principally in the seeds. The naturally occurring forms are four in number: alpha-, beta-, gamma- and delta-tocopherol (a-T, /3-T, 7-T and 6-T). All these forms have been found in foods and fats, although ~-T-3 is rare and, among foods, has been reported only in palm oil [22]. In the same context, argan oil is well known for its richness in tocopherols, particularly γ-tocopherols [23]. It should be noted that prolonged exposure to heat during Soxhlet extraction may potentially affect the stability of tocopherols and may result in some degradation compared with cold mechanical extraction. Therefore, the absolute tocopherol concentrations reported in the present study should be interpreted with consideration of the extraction procedure. According to the results obtained in our study, γ-tocopherols were the most abundant, followed by α-tocopherols, and, finally, δ-tocopherols. These obtained results are in agreement with the majority of published studies [24,25]. Furthermore, the evaluation of the physicochemical quality of *Argania spinosa* fixed oils obtained using Soxhlet apparatus showed that the oil from both genotypes could be classified as extra-virgin according to the Moroccan standard for argan oil quality [26].

The solvent-extracted oils obtained in the present study should not be considered fully representative of commercially consumed argan oil, which is traditionally obtained via mechanical pressing and may involve additional processing steps, such as kernel roasting. Differences in extraction and processing conditions may influence both the oil yield and the composition of minor bioactive compounds. Therefore, the results obtained here primarily reflect the chemical composition of the oil fraction recovered under standardized Soxhlet extraction conditions and should be interpreted accordingly rather than directly extrapolated to commercially produced edible argan oils [24,25].

Also, these results showed that argan oils from three distinct regions were characterized by very promising antioxidant activity. Indeed, several studies have evaluated the antioxidant power of the argan tree, using different methods and different parts of the plant, as well as different extraction methods [27]. The antioxidant potential of the methanolic fraction of argan oil was evaluated in [28], who showed that the antioxidant capacity using the FRAP method is 0.62–2.32 mmoltrolox/kg, while that using the DPPH method is 14.16–28.02 mmoltrolox/kg [29]. On the other hand [30] reported that the IC_50_ of the methanolic fraction of argan oil is 1500 µg/mL using the DPPH method.

The antioxidant power of argan oil could be attributed to the high content of tocopherols, which prevent oxidative damage. In the same way, as indicated by [31], increased oxidative stress is evidenced by elevated lipid peroxidation and heightened DNA damage. Indicators of increased inflammation include elevated monocyte superoxide production and the release of pro-inflammatory cytokines (IL-1, IL-6, and TNF-α), as well as higher levels of plasma C-reactive protein, a prototypic marker of inflammation. Importantly, alpha-tocopherol therapy or high-content tocopherol aliment, especially at high doses, clearly shows benefits regarding LDL oxidation, isoprostanes, and a decrease in inflammatory markers such as C-reactive protein, pro-inflammatory cytokines, and PAI-1 levels.

For better understanding of the underlying mechanism of action of *Argania spinosa*, an in silico approach was conducted for the eight identified compounds. SAR screening and PASS prediction software (https://way2drug.com/PassOnline/; accessed on 10 October 2025), demonstrated that tocopherols have high predicted antioxidant potential (Pa = 0.843–0.967); however, fatty acids showed moderate scores (Pa = 0.222–0.314). These results corroborate the reported findings in the literature, which refers the antioxidant properties of *Argania spinosa* to the existence of a high amount of tocopherols [23]. The elaborated computational investigation indicated that fatty acids exhibited higher insulin-promoting effects that tocopherols, indicating a complimentary system in which fatty acids influence glycemic regulation via enzymatic pathways while tocopherols function as direct radical scavengers. This could be attributed to the long-chain fatty acids that are known for their ability to enhance insulin secretion through the FFAR1/GPR40 signaling pathway. The Protein-Coupled Receptor 40 (GPR40), another name for Free Fatty Acid Receptor 1 (FFAR1), is a receptor that is mostly expressed in pancreatic β cells. It is essential for glucose-stimulated insulin production and is activated by medium- and long-chain fatty acids. When FFAR1/GPR40 is activated, it links to Gq protein, which activates phospholipase C, raises intracellular calcium levels, and amplifies the release of insulin. Because of this process, fatty acids can function as metabolic signals that improve glycemic control by increasing insulin secretion in a glucose-dependent way [32,33].

Furthermore, 51 common genes with antioxidant, antidiabetic, and *Argania spinosa* annotations were found through target integration, with metabolic–inflammatory pathways such as AGE-RAGE signaling, insulin resistance, and NAFLD (q ≤ 10^−2^) being enriched. Since AGEs cause diabetes problems due to irreversible crosslink formation, the AGE-RAGE result mechanistically supports our reported antiglycation activity (IC_50_: 0.09–0.77 mg/mL). While PPI mapping verified abundant PPARG-mediated linkages between signaling and metabolic proteins, suggesting tocopherol-fatty acid synergy, GO analysis focused on lipid metabolism and reactions to chemical/hormonal stimuli (q ≤ 10^−11^). According to molecular docking, tocopherols have strong binding energy to glycemic and antioxidant targets: α-tocopherol led for iNOS (−9.3 kcal mol^−1^) and α-glucosidase (−8.2), while α/γ-tocopherol outperformed acarbose and trolox for α-amylase (−8.6/−8.3). The binding of fatty acids was milder (−6.6 to −4.8). According to previous reports on fatty acids and α-tocopherol therapy [34], δ-tocopherol’s glucosidase binding supports tocopherol-rich oils as carbohydrate enzyme inhibitors for diabetes management [35], which explains our finding that α-tocopherol-rich Chouihya oil had the lowest α-amylase IC_50_.

Comparing the antidiabetic activity of the oils between regions, it was found that Chouihya argan oil had the lowest IC_50_ for α-amylase inhibition, followed by Agadir oil and finally Oujda oil, and according to the statistical correlation analysis, α-amylase inhibitory power is negatively correlated with alpha-tocopherol content, meaning that the higher the tocopherol content, the lower the IC_50_, which is justified since argan oil from Chouihya is the richest in α -tocopherols. This has been confirmed by several studies, which indicate that α-tocopherols are characterized by significant antidiabetic activity by different pathways [36,37] Concerning the inhibition of hemoglobin glycation, it was shown that argan oil from Oujda was the most potent and had similar activity to that recorded by gallic acid with no statistically significant difference, followed by that from Chouihya and finally oil from Agadir. According to the chemical composition analysis, the oil from Oujda was the richest in γ-tocopherol and δ-tocopherol and represented a high level of palmitic, oleic and linoleic acids.

Concerning the antidiabetic activity of argan oil assessed by targeting two key proteins in the prevention of diabetes, our results are in agreement with those found by [4], where it was reported that α-amylase inhibition had an IC_50_ of 2.17 mg/mL for oil extracted from roasted kernels and an IC_50_ of 0.78 mg/mL obtained with oil from unroasted kernels. This might be explained by the oil’s richness in fatty acids, which are known to have a significant antidiabetic effect. Indeed, it has been recorded that two fatty acids present in our oils, palmitic acid and linoleic acid, have a weak to moderate ability to inhibit pancreatic α-amylase [38]. The inhibition of pancreatic α-amylase is a targeted pathway for the treatment of diabetes mellitus [39]. In addition, the stronger α-amylase inhibitory activity observed for Chouihya oil was associated with its particular chemical profile, including its tocopherol composition. However, this association does not establish a causal contribution of α-tocopherol or any individual compound. The observed inhibitory activity may instead result from the combined contribution of multiple oil constituents and their potential interactions [38,39].

The study conducted by [4] showed a comprehensive comparison of the antioxidant and hemoglobin antiglycation activities of roasted and unroasted argan seed oil, in vitro. Therefore, the hemoglobin glycation assay showed that both roasted and unroasted argan seed oil exhibited an antiglycation effect, with IC_50_ values of 1.09 ± 0.31 and 0.16 ± 0.031 mg/mL, respectively. In comparison to our study, the IC_50_ values vary between 0.09 ± 0.01 mg/mL and 0.77 ± 0.006 mg/mL, and this was in the same line to the results found by [4].

The antidiabetic activity could be attributed to the tocopherols present in argan oil. The study performed by [22] indicated that α-tocopherol decreases glycated HbA1c and pancreatic lipid peroxidation in rats. In the same way, in a study by [36], GK rats were fed a diet containing 0, 20 or 500 mg/kg α-tocopherol; the results showed a significant decrement in blood glucose levels at 30 and 120 min after glucose loading, and the levels of glycated hemoglobin HbA1c were also reduced. Furthermore, ref. [33] indicated that in diabetes, alpha-tocopherol therapy could potentially emerge as an additional therapeutic modality.

The glycation process, also known as the Maillard reaction, begins with the condensation of free amino groups in proteins with the carbonyl groups of reducing sugars, leading to the formation of reversible Schiff base compounds [40]. These compounds undergo further rearrangement into more stable Amadori products, which serve as precursors to a cascade of complex chemical reactions. Over time, these reactions give rise to advanced glycation end products (AGEs), which are highly reactive and form irreversible, complex molecular structures. AGEs can interact with nearby amino groups of proteins, lipids, and nucleic acids, leading to the formation of intra- and intermolecular crosslinks [41]. The accumulation of AGEs has been implicated in aging and the progression of various chronic diseases, including diabetes, cardiovascular disorders, and neurodegenerative conditions, due to their ability to induce oxidative stress and inflammation [41,42].

The impact of natural product bioactive compounds such as tocopherols and fatty acids, including oleic and linoleic acids, on the glycation of glucose has been widely studied due to their potential antioxidant and structural effects [43] Tocopherols, known for their strong antioxidant properties, can inhibit glycation by neutralizing reactive oxygen species (ROS) that accelerate the formation of advanced glycation end products (AGEs) [44]. By reducing oxidative stress, tocopherols help prevent the modification of proteins and lipids, thereby mitigating the harmful consequences of glycation. Similarly, unsaturated fatty acids such as oleic and linoleic acids have been shown to modulate glycation through their ability to influence membrane fluidity and cellular metabolism. Oleic acid, a monounsaturated fatty acid, exhibits protective effects by enhancing antioxidant defenses and reducing lipid peroxidation, which indirectly curbs AGE formation [45]. Linoleic acid, a polyunsaturated fatty acid, can also impact glycation by modulating inflammatory responses and oxidative pathways [45].

Omega-3 (ω-3) and omega-6 (ω-6) fatty acids (FAs) are two classes of essential dietary polyunsaturated fatty acids (PUFAs), derived, respectively, from α-linolenic acid (ALA, 18:3 ω-3) and linoleic acid (LA, 18:2 ω-6) [45,46]. The study conducted by [45] reported the protective effects of linolenic acid towards the formation of early HbA1c.

Our in silico pharmacological network offers a deep molecular understanding of the conducted experimental findings. The identified δ-tocopherol from argan oil may act as a major multi-target contributor to the obtained pharmacological effects, such as antioxidant and antidiabetic, while fatty acids may contribute through complementary effects involving enzymatic and insulin-related pathways. These proposed contributions require further experimental validation. The strong antiglycation action found in all three argan oil genotypes is directly supported by the enrichment of the AGE-RAGE signaling pathway in our computational investigation.

## 4. Materials and Methods

### 4.1. Preparation of Argania spinosa Oils

**Figure 7:**
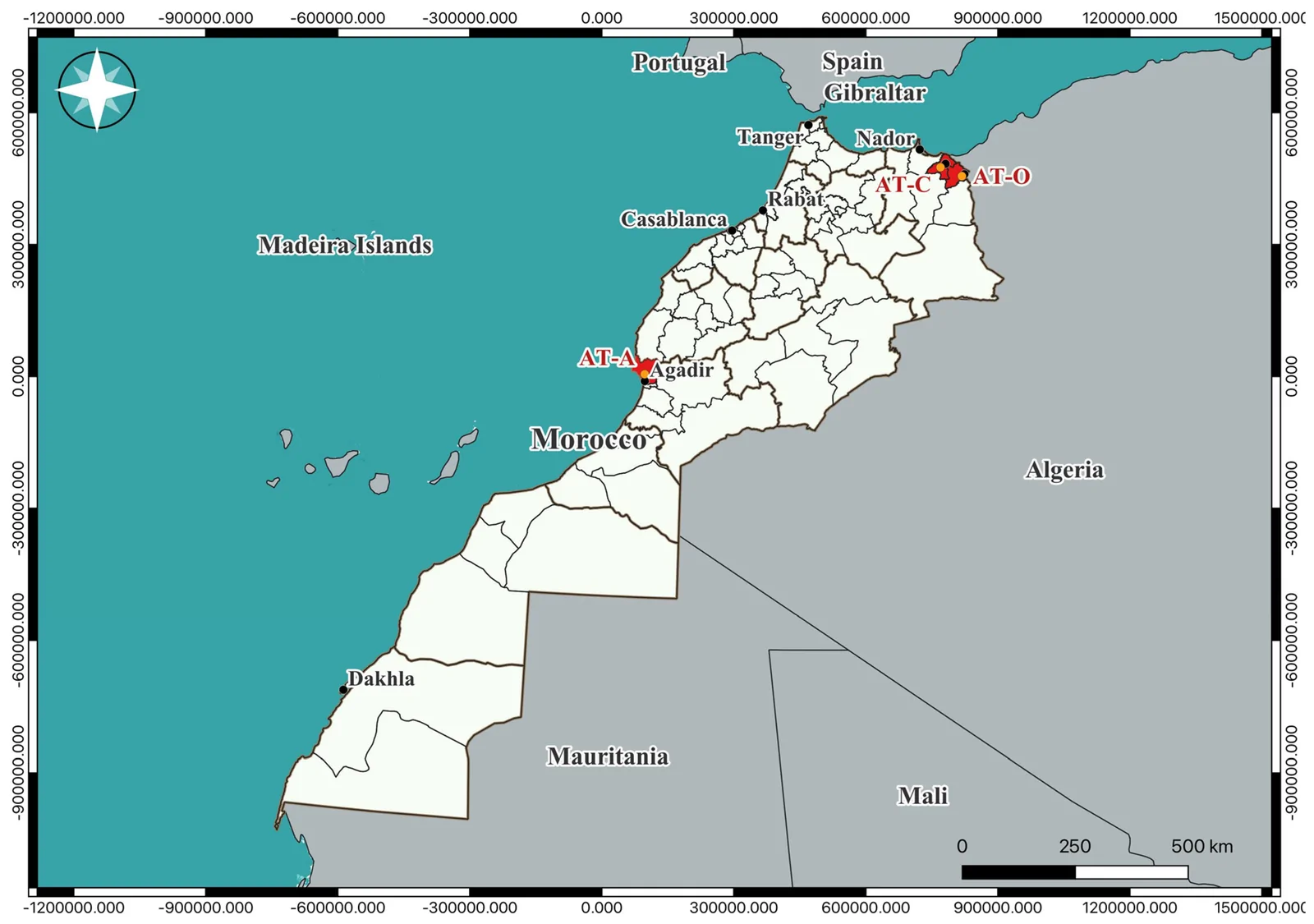
Geographic location of the argan tree accessions used in this study. Trees were selected based on agronomic, pomological, and oil-quality criteria, as previously described in [9] (AT-O, AT-C, and AT-A).

Argan fruits were collected from three selected argan trees, representing three different geographical origins. The first tree, designated AT-O, was native to Oujda, while the second tree, designated AT-C, was indigenous to Chouihya. Both trees were selected based on agronomic and phytochemical criteria established in our previous study [10], particularly their high fruit and oil yields. For comparison, argan oil obtained from a tree native to Agadir was included as a reference and designated AT-A. For each tree, mature fruits were collected and processed separately to obtain the corresponding argan oil. The oil obtained from each individual tree was considered an independent biological sample. The three oil samples (AT-O, AT-C, and AT-A) were subsequently subjected to different experimental assays. Each assay was performed in three technical replicates, and the results are expressed as mean ± standard deviation (Figure 7).

### 4.2. Extraction Process of Argania spinosa Oil

Prior to oil extraction, the kernels were placed in an oven for 24 h at a temperature of 35 °C. Next, 50 g of the dried kernels was transformed into a fine powder that was subjected to chemical extraction in Soxhlet apparatus using n-Hexane (250 mL) as extraction solvent. The extraction process lasted around three hours until the plant material was completely exhausted. A rotary evaporator was then employed to completely remove the extraction solvent. Once recovered, the oils were placed in 30 mL opaque glass vials and stored at −20 °C until further use.

### 4.3. Chemical Composition Analysis of Argania spinosa Fixed Oil

#### 4.3.1. Argan Oil Tocopherol Analysis Using HPLC-DAD

The different types of tocopherols (α, δ, γ) were separated and identified using the methods described by [47]. The oils were dissolved in hexane (0.5/0.5 *w*/*w*) and analyzed using HPLC apparatus (Shimadzu LC-6AD system) equipped with a DAD detector. Tocopherols were separated using the NH2 Uptisphere 120 Å column (150 mm × 3 mm, 3 m) Interchim (Montluçon, France), the solvent system used was hexane/2-propanol (99:1, *V*/*V*) eluted at a flow rate of 1 mL/min, and the injection volume was 20 L. Identification was carried out using commercial standards for tocopherols (Sigma-Aldrich, St-Louis, USA) at 292, 296 and 298 nm. Tocopherol concentration was calculated from the external calibration curve with commercial tocopherols obtained from Sigma-Aldrich (St-Louis, MO, USA) [48] All experiments were performed in triplicate, and data were expressed as mean ± SD.

#### 4.3.2. Analysis of Argan Oil Methyl Esters by GC-MS

The preparation of methyl esters was assessed following the NF T60-233 standard protocol [49]. The esterified hexanic extracts were subjected to chemical analysis using gas chromatography (Shimadzu GC-2010) coupled with a mass spectrometer detector (GC-MS-QP2010) via a fused silica capillary column. Helium pressure was adjusted to 100 kPa, and the oven temperature was programmed to increase from 50 °C (held for 1 min) at a rate of 10 °C/min until reaching 250 °C (held for 1 min). For qualitative and semi-quantitative analysis, 1 µL of the original samples diluted in hexane (50 mg/g) was injected in split mode (split ratio = 50–80). Mass spectra were acquired at 70 eV (electron impact ionization mode) within an *m*/*z* range of 40–350 a.m.u. (with velocity and solvent delays set at 5 s/scan and 4.5 min, respectively). Peak identification was performed by comparing the MS data with the National Institute of Standards and Technology library (NIST 147). LabSolutions (version 2.5) was utilized for data collection and processing.

The GC-MS analysis was performed qualitatively and semi-quantitatively. The relative abundance of each identified fatty acid methyl ester was calculated from its chromatographic peak area and expressed as a percentage of the total peak area of the identified FAMEs. Therefore, the reported percentages represent relative peak-area contributions and do not correspond to absolute fatty acid concentrations.

#### 4.3.3. Assessment of the Physicochemical Quality of Argan Oil

The acid value and peroxide value were assessed following Codex standard 210-1999 [49] nd following the Moroccan standard for argan oil (NM 08.5.090) [49]. Specific extinction was determined using the official analytical method ISO 3656:2002 [50]. All experiments were conducted in triplicate, and the results were presented as mean ± SD.

### 4.4. In Silico Study Analysis

#### 4.4.1. Prediction of Phytochemical Activities and Pharmacokinetic Properties

Bioactivities of argan oils were estimated with WAY2DRUG PASS prediction tool (https://way2drug.com/PassOnline/; accessed on 10 October 2025), which applies SAR modeling to return a Probability of Activity (PA) for predefined endpoints [51,52]. We screened at PA > 0.5 (with PA > 0.7 considered highly suggestive). Analyses were run on 10 October 2025. Pharmacokinetic and toxicological properties (ADMET) were profiled using ADMET LAB 3.0 and Protox III [53,54]. Drug-likeness was assessed by Lipinski’s Rule of Five. Canonical SMILES for each compound were retrieved from PubChem and used across platforms [55].

#### 4.4.2. Prediction and Functional Analysis of Protein Targets

Computational target profiling of argan oil constituents was performed using SuperPred (https://prediction.charite.de/; accessed on 10 October 2025 [56]. For each compound, SMILES strings were submitted, and predicted targets with scores ≥ 80% (0–100% scale) were retained. Disease-associated genes/proteins for antioxidant and antidiabetic were retrieved from Gene Cards (https://www.genecards.org/; accessed on 10 October 2025) [56]. Overlap between predicted compound targets and disease gene sets was assessed by Venn analysis (https://bioinformatics.psb.ugent.be/webtools/Venn/; accessed on 10 October 2025).

#### 4.4.3. Network Pharmacology Analysis

We analyzed protein–protein interaction (PPI) networks to map how targets derived from *Argania spinosa* oils may relate to antioxidant and antidiabetic activities. Biological pathway enrichment for these targets was evaluated with ShinyGO (v0.85) to identify KEGG pathways significantly associated with the protein set (https://bioinformatics.sdstate.edu/go/; accessed on 10 October 2025) [57]. PPIs were assembled with STRING (v12.0), where targets from *Argania spinosa* extracts were intersected with protein datasets linked to antioxidant and antidiabetic activities to define a combined network [39]. Analyses were restricted to *Homo sapiens* and limited to high-confidence edges (interaction score ≥ 0.9) to retain only robust associations. Interaction tables were exported from STRING as TSV files for downstream processing. Network topology was then examined in Cytoscape (v3.10.1), enabling detailed assessment of key graph properties such as node degree and inter-receptor connectivity patterns [56].

#### 4.4.4. Computational Docking and Protein–Ligand Interaction Profiling

Blind docking was carried out with CB-Dock2, the upgraded CB-Dock platform that integrates CurPocket for curvature-based cavity detection, AutoDock Vina V 1.2.0 for docking, and homologous template fitting [34,58]. CB-Dock2 streamlines the workflow by automatically locating putative pockets, estimating their geometric parameters, sizing the search box to each ligand, and executing the docking runs—an integration reported to enhance both convenience and predictive performance [59]. We prioritized receptors with the highest network centrality, especially those embedded in key signaling pathways, for detailed docking analysis. Protein structures were retrieved from the RCSB PDB on 10 October 2025 (iNOS:3E7G; α-Amylase:2QV4; α-Glucosidase:3L4Y; TCFL72:1JDH) and, prior to docking, the CB-Dock server removed water molecules and other heteroatoms [59]. Ligands were obtained from PubChem in SDF format on the same date; for compounds not available there, structures were drawn in ChemDraw 22.2.0 [55].

### 4.5. In Vitro Study

#### 4.5.1. Antiradical Scavenging Activity Against DPPH•

From each extract, eight concentrations were prepared (from 40 to 800 µg/mL). Thus, 200 mL of each extract was mixed with 1.8 mL of DPPH methanolic solution (4 mg/100 mL). The mixture was then incubated for 30 min in the dark and at room temperature for 20 min. Absorbance was measured at 517 nm against blank. Ascorbic acid was used as the standard. All measurements were performed in triplicate [57,60].

The percentage is calculated using the following equation:

$$Inhibition\;(\%)=\frac{\left({Abs}_{control}-{Abs}_{sample}\right)}{{Abs}_{control}}\times 100$$

Ferric reducing power (FRAP):

One volume of each extract (0.5 mL) was mixed with 1.25 mL phosphate buffer (0.2 M, pH = 6.6) and 1.25 mL potassium ferricyanide [K_3_Fe (CN)_6_] (1% *w*/*v*). The mixture was incubated at 50 °C for 20 min. After cooling to room temperature, the reaction was stopped by adding 1.25 mL trichloroacetic acid (10% *w*/*v*). After centrifuging the mixture at 3000 rpm for 10 min, 1.25 mL of the supernatant was mixed with 1.25 mL of bi-distilled water and 0.25 mL of ferric chloride (FeCl_3_) (0.1% *w*/*v*). Absorbance was measured at 700 nm against a blank containing bi-distilled water. Ascorbic acid was used as a reference. All measurements were performed in triplicate [57,61].

#### 4.5.2. Evaluation of the In Vitro Inhibitory Activity of α-Amylase

The inhibitory capacity of the different extracts and oils on the pancreatic α-amylase enzyme was examined according to the method described by [62,63]. The reaction mixture contained 200 µL of the oil or acarbose used as a control at different concentrations, 200 µL of phosphate buffer (pH = 6.9) and finally 200 µL of the α-amylase enzyme solution. The reaction mixture was pre-incubated at 37 °C for 10 min. Then, 200 µL of starch (1%) was added to each tube and incubated at 37 °C for 20 min. To stop the reaction, 600 µL of 3,5-dinitrosalicylic acid (DNSA) was added to each tube. All tubes were then incubated at 100 °C for 8 min and cooled in a cold-water bath. Finally, the tubes containing the fractions and the control were diluted with 1 mL of distilled water. The absorbance of the different samples was measured at 540 nm.

Percentage inhibition was calculated using the following formula:

$$Inhibition\;(\%)=\frac{\left({OD}_{control}-{OD}_{sample}\right)}{{OD}_{control}}\;\times 100$$

#### 4.5.3. Evaluation of the In Vitro Hemoglobin Glycation Inhibition

The evaluation of argan oil’s ability to inhibit hemoglobin glycation was carried out according to the protocol described by [38]. In a buffered medium (phosphate buffer pH = 7.4), a 1 mL volume of the 5% hemoglobin solution was added to 5 µL gentamicin and 25 µL of the argan oil or gallic acid at different concentrations ranging from 0.06 mg/mL to 1 mg/mL. Next, 1 mL of glucose (4 mg/mL) was added to the mixture to trigger the reaction. The reaction mixture was incubated for 72 h in the dark at room temperature, and absorbance was determined spectrophotometrically at 443 nm.

Inhibition percentage was calculated using the formula below:

$$Inhibition\;(\%)=\frac{\left({OD}_{control}-{OD}_{blank}\right)-({OD}_{sample}-{OD}_{sample\;blank})}{{OD}_{control}-{OD}_{control\;blank}}\times 100$$

### 4.6. Statistical Analysis

Study results were subjected to descriptive statistical analysis and analysis of variance (ANOVA), using “SPSS for Windows version 23” software followed by Tukey’s test with a post hoc multiple comparison threshold of 5%. Pearson correlation was assessed using the same software. Three independent argan oil samples (n = 3) were considered as independent experimental observations. Each sample was analyzed in triplicate as technical/analytical replicates, and the technical replicates were used to calculate the mean ± standard deviation (SD) for each independent sample. Technical replicates were not considered independent biological replicates.

Given the limited number of independent samples (n = 3), formal tests of normality and homogeneity of variance have limited statistical power and were, therefore, interpreted cautiously; these assumptions cannot be considered robustly verified from such a small number of independent observations. Accordingly, statistical significance was assessed at a threshold of *p* < 0.05. Statistical differences were interpreted according to the significance levels provided by the statistical software.

Pearson correlation analysis was performed using the same software. Because the correlation analysis was based on only three independent observations (n = 3), it was considered exploratory and descriptive. The resulting correlation coefficients were, therefore, interpreted cautiously and were not considered evidence of robust or generalizable associations.

## 5. Conclusions

In conclusion, the results obtained showed that the argan oil produced by trees introduced in eastern Morocco is characterized by a nutritional quality equivalent to that produced by argan trees growing in their natural habitat, particularly in southwestern Morocco. Additionally, in northeastern Morocco, they were characterized by interesting antioxidant and antidiabetic activities, notably their ability to inhibit alpha-amylase and hemoglobin glycation. These findings support further investigation of argan oil bioactive compounds as potential functional food components.

## Figures and Tables

**Table 1:** Chemical composition and physicochemical parameters of *Argania spinosa* L. oils obtained from trees originating from Oujda, Agadir, and Chouihya, Morocco. Fatty acid composition was determined by GC-MS, while tocopherol content was determined by HPLC-DAD using authentic reference standards. Physicochemical parameters were determined according to the corresponding analytical methods described in Section 4. Values are expressed as mean ± standard deviation.

Chemical Composition		Argan Oil Oujda	Argan Oil Agadir	Argan Oil Chouihya
Fatty acids %	Palmitic acid	14.41 ± 0.01	13.58 ± 0.02	9.97 ± 0.04
	Linoleic acid	30.54 ± 0.01	26.75 ± 0.12	31.38 ± 0.07
	Oleic acid	49.31 ± 0.02	47.57 ± 0.07	51.58 ± 0.11
	Stearic acid	5.73 ± 0.02	12.09 ± 0.05	7.04 ± 0.04
Tocopherols mg/kg	α-tocopherol	41.83 ± 0.39	42.59 ± 4.19	57.69 ± 3.01
	γ-tocopherol	447.66 ± 4.54	373.73 ± 6.7	446.03 ± 14.79
	δ-tocopherol	37.74 ± 1.53	36.65 ± 0.83	28.22 ± 1.55
	Total tocopherols	527.24 ± 6.48	452.99 ± 11.73	531.94 ± 19.37
*Physicochemical parameters*	K232	1.10 ± 0.24	1.36 ± 0.06	1.03 ± 0.08
	K270	0.11 ± 0.00	0.12 ± 0.005	0.18 ± 0.01
	ΔK	0.00 ± 0.00	0.00 ± 0.00	0.00 ± 0.00
	Free acidity (%)	0.27 ± 0.00	0.64 ± 0.01	0.54 ± 0.00
	Peroxide value	3.30 ± 0.15	1.27 ± 0.09	1.50 ± 0.08

**Table 2:** GC–MS-identified compounds and their molecular characteristics in the extract.

No.	Name	Molecular Formula	PubChem/CAS ID	Calculated/Observed MW (g/mol)
C1	Palmitic acid	C_16_H_32_O_2_	985	256.42
C2	Linoleic acid	C_18_H_32_O_2_	5280450	280.4
C3	Oleic acid	C_18_H_34_O_2_	445639	282.5
C4	Stearic acid	C_18_H_36_O_2_	5281	284.5
C5	α-tocopherol	C_29_H_50_O_2_	14985	430.7
C6	γ-tocopherol	C_28_H_48_O_2_	92729	416.7
C7	δ-tocopherol	C_27_H_46_O_2_	92094	402.7

**Table 3:** Predicted antioxidant and antidiabetic activities of *Argania spinosa* supported by SAR, PaScore, toxicity/drug-likeness filters, and network pharmacology.

Compounds	Pa Score *						Toxicity Model Computation Analysis **		Drug-Likeness ***		
	Antioxidant	Superoxide Dismutase (SOD) Inhibitor	Catalase Inhibitor	Alpha-Amlylase Inhibitor	Beta-Amylase Inhibitor	Insulin Promoter	Predicted LD_50_ (mg/kg)	Toxicity Class	Lipinski Rule	Pfizer Rule	GSK
C1	0.222	0.914	0.819	0.583	0.593	0.753	900	4	Accepted	Rejected	Rejected
C2	0.314	0.814	0.712	0.490	0.301	0.597	10,000	6	Accepted	Rejected	Rejected
C3	0.283	0.851	0.749	0.514	0.372	0.648	48	2	Accepted	Rejected	Rejected
C4	0.222	0.914	0.819	0.583	0.593	0.753	900	4	Accepted	Rejected	Rejected
C5	0.967	NA	NA	NA	NA	NA	5000	5	Accepted	Rejected	Rejected
C6	0.927	NA	NA	NA	NA	0.278	5000	5	Accepted	Rejected	Rejected
C7	0.843	NA	NA	NA	NA	0.306	5000	5	Accepted	Rejected	Rejected

“NA: not applicable. * Predicted by PASS software. ** Predicted by ProTox-II. *** Assessed via Lipinski, Pfizer, and GSK rules using SwissADME.”

**Table 4:** ΔG values from molecular docking of metabolites related to antioxidant and antidiabetic activity from *Argania Spinosa*.

Samples and Control	Observed Compounds as Ligands	iNOS (3E7G)	α-Amylase (2QV4)	α-Glucosidase (3L4Y)	TCF7L2 (1JDH)
Control	Trolox	−8.5	−7.5	−6.8	−6.2
Control	Acarbose	−7.8	−7.6	−7.0	−6.8
Fatty Acids	Palmitic acid	−6.6	−5.5	−6.2	−5.4
	Linoleic acid	−7.0	−5.6	−6.1	−4.8
	Oleic acid	−6.8	−6.1	−5.7	−5.1
	Stearic acid	−6.7	−5.3	−6.0	−5.1
Tocopherols	α-tocopherol	−8.3	−8.6	−7.7	−6.0
	γ-tocopherol	−8.7	−8.3	−7.7	−5.9
	δ-tocopherol	−9.3	−8.4	−8.2	−5.7

**Table 5:** Recorded IC_50_ value in mg/mL of free radical scavenging activity of *Argania spinosa* fixed oils against stable radical DPPH•.

*Argania spinosa* Fixed Oils	Oil: Oujda	Oil: Chouihya	Oil: Agadir	Ascorbic Acid
IC_50_ (mg/mL)	15.25 ± 0.022 a	17.17 ± 0.018 c	16.71 ± 0.02 b	0.021 ± 0.007 d

Values followed by different letters are significantly different (*p* < 0.05); values with the same letters indicate no significant difference (*p* > 0.05).

**Table 6:** Recorded IC_50_ value in mg/mL of ferric reducing power of *Argania spinosa* fixed oils.

*Argania spinosa* Fixed Oil	Oil: Oujda	Oil: Chouihya	Oil: Agadir	Ascorbic Acid
IC_50_	28.5 ± 1.7 a	34.2 ± 0.28 c	31.3 ± 0.75 b	0.06 ± 0.001 d

Values followed by different letters are significantly different (*p* < 0.05); values with the same letters indicate no significant difference (*p* > 0.05).

**Table 7:** IC_50_ value of *Argania spinosa* oil α-amylase inhibition assay.

*Argania spinosa* Fixed Oil	Oil: Oujda	Oil: Chouihya	Oil: Agadir	Acarbose
IC_50_	3.66 ± 0.56 ***	0.65 ± 0.28 ^NS^	0.70 ± 0.18 ^NS^	0.74 ± 0.002

NS = not significant (*p* > 0.05), *** = Highly significant (*p* < 0.001).

**Table 8:** IC_50_ of hemoglobin glycation inhibition by *Argania spinosa* fixed oils.

*Argania spinosa* Fixed Oil	Oil: Oujda	Oil: Chouihya	Oil: Agadir	Gallic Acid
IC_50_	0.09 ± 0.01 ^NS^	0.49 ± 0.12 ***	0.77 ± 0.006 ***	0.07 ± 0.01

NS = not significant (*p* > 0.05), *** = Highly significant (*p* < 0.001).

**Table 9:** Statistical correlation between the chemical composition of oils and target proteins.

	Palmitic Acid	Linoleic Acid	Oleic Acid	Stearic Acid	α-Tocopherol	γ-Tocopherol	δ-Tocopherol	T-Tocopherol
IC_50_ α-amylase	0.630	0.361	−0.059	−0.672	−0.523	0.531	0.160	0.468
IC_50_ HBA1C	−0.902	0.074	0.482	0.289	0.839	−0.115	0.280	−0.043
IC_50_ DPPH	−0.948	0.194	0.584	0.171	0.898	0.005	0.393	0.078
IC_50_ FRAP	−0.942	0.177	0.570	0.188	0.891	−0.012	0.378	0.061

## Data Availability

The original contributions presented in this study are included in the article/Supplementary Materials. Further inquiries can be directed to the corresponding author.

## Supplementary Materials

The following supporting information can be downloaded at: https://www.mdpi.com/article/10.3390/ijms27177964/s1.
