# Supplementary material for: Domesticated Argania spinosa in Eastern Morocco: HPLC-DAD/GC-MS Chemical Profiling, Antioxidant and Antidiabetic Activities, and Network Pharmacology-Guided Molecular Docking
Source: Int J Mol Sci. 2026 Sep 7;27(17):7964. doi: 10.3390/ijms27177964 (PMC13566589; doi:10.3390/ijms27177964)
Supplement: Supplementary file 1 [file ijms-27-07964-s001.zip › ijms-4462979-supplementary.pdf]

Supplementary materials:

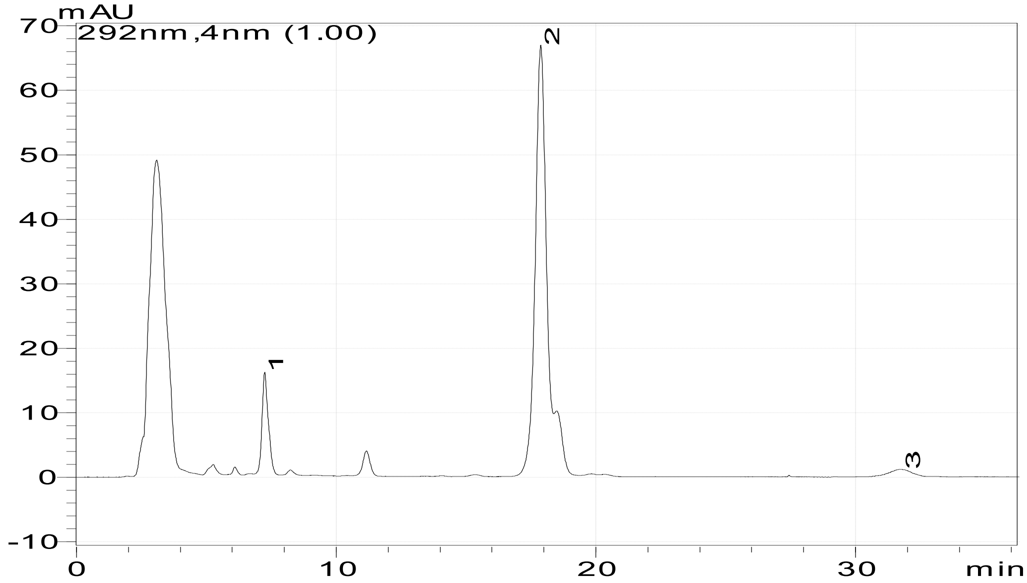

Figure S1 : **HPLC-DAD chromatogram obtained for the analyzed argan oil**

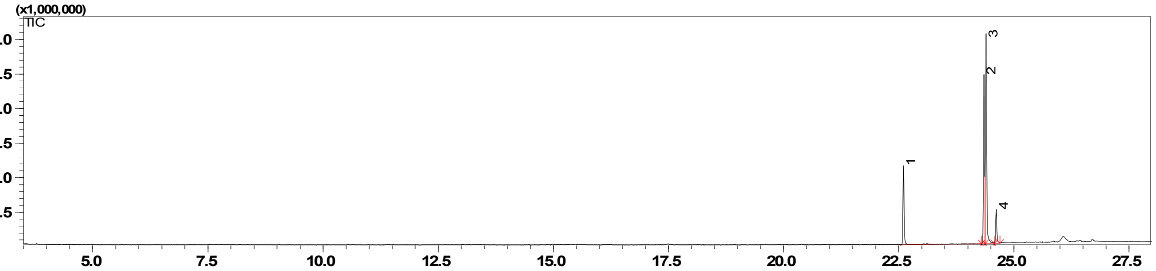

Figure S2. : **CPG-MS chromatogram obtained for the analyzed argan oil**
